# Causal mediation analysis of a randomised controlled trial in China: evaluating whether the pay-it-forward strategy increases HPV vaccine uptake by reducing vaccine delay intention and increasing vaccine confidence

**DOI:** 10.1136/bmjopen-2024-095248

**Published:** 2025-09-25

**Authors:** Yajiao Lu, Ying Yang, Yifan Li, Chuanyu Qin, Yu He, Wenfeng Gong, Shenglan Tang, Jing Li, Dan Wu

**Affiliations:** 1 West China School of Public Health and West China Fourth Hospital, Sichuan University, Chengdu, China; 2 Department of Social Medicine and Health Education, Nanjing Medical University School of Public Health, Nanjing, China; 3 Yulin Community Health Service Center, Chengdu, China; 4 Bill & Melinda Gates Foundation China, Beijing, China; 5 Duke University, Duke University Global Health Institute, Durham, North Carolina, USA

**Keywords:** Vaccination, China, Community-Based Participatory Research, Immunization Programs, HPV Vaccine, Parents

## Abstract

**Objective:**

To explore whether vaccine confidence and vaccine delay intention mediated the effect of the pay-it-forward intervention on human papillomavirus (HPV) vaccine uptake.

**Design:**

This secondary mediation analysis of a two-arm randomised controlled trial was conducted among female adolescents aged 15–18 years in Chengdu, China, from July 2022 to June 2023.

**Setting:**

This study was conducted in four residential areas representing diverse economic backgrounds in Chengdu.

**Participants:**

A total of 321 parents of girls aged 15–18 years who had not received the HPV vaccine participated in the study.

**Intervention:**

Participants were randomly allocated into two arms. Pay-it-forward participants received a community-contributed subsidy (47.7 USD) to support the HPV vaccination, along with educational postcards and an opportunity to donate to support others. In the standard-of-care arm, participants paid for their vaccination.

**Primary and secondary outcome measures:**

Primary outcome was the receipt of the first HPV vaccine dose within a 3-month period following an intervention. Based on previous literature, we hypothesised that vaccine confidence and vaccine delay intention were potential mediators. Vaccine confidence was measured using the vaccine confidence index. Vaccine delay intention refers to the caregiver’s preference to postpone HPV vaccination for their daughter until the preferred vaccine type becomes available, rather than accepting the immediately accessible HPV vaccine. Data on these mediators were collected via a self-administered online questionnaire conducted after the intervention but before vaccination.

**Results:**

Among urban participants, when compared with the standard-of-care arm, about 39% of the effect of the pay-it-forward intervention on vaccine uptake was mediated by a reduction in vaccine delay intention. Notably, vaccine confidence did not appear to mediate the effect of the intervention on vaccine uptake. Among suburban participants, no mediation effects were observed. In the suburban setting, caregivers who vaccinated their daughters showed poorer prior awareness of the HPV vaccine before participating in the trial compared with those who did not vaccinate their daughters (41.5% vs 21.1%; p=0.011).

**Conclusion:**

Our findings indicate that among urban participants, the pay-it-forward may have effectively reduced vaccine delay intention, which was associated with an increased uptake of the HPV vaccine. However, in suburban areas, enhanced awareness might be a potential contributing factor to improved vaccine uptake, but further research is necessary to affirm this.

**Trial registration number:**

Chinese Clinical Trial Registry: ChiCTR2200055542.

**STRENGTHS AND LIMITATIONS OF THIS STUDY**

- The analysis is based on a high-quality randomised controlled trial.
- Causal mediation analysis is usually preferred because it can accommodate more realistic settings, such as non-linear relations.
- The randomised controlled trial was not originally designed for mediation analysis, and the mediators were pre-identified and measured based on a limited range of potential factors.

## Introduction

 Cervical cancer poses a significant global health challenge, with China carrying a particularly heavy burden. In 2020, an estimated 110,000 new cases and 59,000 fatalities were attributed to cervical cancer in China.^1^ Most cervical cancers result from persistent infections with high-risk human papillomaviruses (HPVs).^2^ HPV vaccination is the most cost-effective preventive measure.^3^ Previous research has shown that a majority of Chinese parents (61.0%) express a willingness to vaccinate their children against HPV.^4^ However, despite this high willingness, fewer than 5% of females <20 years old have received the first dose of the vaccine.^5^

Several factors contribute to the low vaccination rate. First, HPV vaccination is not included in China’s national immunisation programme, and the associated fees can prohibit many from vaccination. As of 2023, five types of HPV vaccines are available in the Chinese market: Cecolin (domestic 2v-HPV), Walrinvax (domestic 2v-HPV), Cervarix (imported 2v-HPV), Gardasil (4v-HPV) and Gardasil9 (9v-HPV). But there are frequent stockouts of 4v-HPV and 9v-HPV vaccines all over the country, and domestic 2v-HPV vaccines lack market demand.^6^ Lack of vaccine confidence in alternative domestic products^7 8^ and vaccine delay intention^9 10^ are common. Information overload and absence of tailored campaigns further complicated the issue. Innovative vaccination messaging and incentive models are needed to address these barriers.

The pay-it-forward intervention encourages healthy behaviours and community contributions. It gives one person a subsidised HPV vaccine along with a handwritten postcard as a gift, followed by voluntary donation and/or creation of a postcard message to support another person. Our previous two-arm randomised controlled trial (RCT) suggests that this model can improve earlier uptake of available HPV vaccines compared with standard-of-care self-paid vaccination arm.^11^ However, our understanding of the underlying mechanisms driving this success is limited. Identifying potential mediating variables is crucial to further optimising the intervention’s effectiveness. Causal mediation analysis uses a counterfactual framework to define direct, indirect and total effects, and can help clarify causal assumptions.^12^,^14^ In this analysis, we hypothesised that pay-it-forward might have an impact on vaccine confidence and vaccine delay intention, which might be associated with an increased HPV vaccine uptake. The mediation analysis aims to test these hypotheses. Given participants from urban and suburban areas might have substantial differences in socioeconomic and health literacy backgrounds, we stratified the analyses by urban and suburban contexts.

## Methods

### Study design

This study adheres to the AGReMA (A Guideline for Reporting Mediation Analyses).^15^ It is a secondary causal mediation analysis based on the results from a two-arm RCT that evaluated the effects of the pay-it-forward intervention on HPV vaccine uptake. Detailed descriptions of the trial design, study setting, recruitment, randomisation and intervention were previously published elsewhere.^16^ The original trial was prospectively registered in the Chinese Clinical Trial Registry (ChiCTR2200055542) and approved by the Ethics Committee of West China Fourth Hospital and West China School of Public Health (Gwll2021057/Gwll2023125). Verbal consent was obtained from caregivers during telephone recruitment, followed by written informed consent at enrolment, with adolescent girls providing assent at the on-site visit. This secondary analysis used a fully de-identified dataset. Exploration of the pay-it-forward intervention mechanisms was specified in the original ethics application, and the initial consent process informed participants that anonymised data could be used for subsequent analyses and publications related to the study.

### Population and inclusion criteria

This randomised clinical trial was conducted among female adolescents aged 15–18 years in Chengdu, China, from 6 July 2022 to 9 June 2023. Four residential areas representing diverse economic backgrounds (high-income, middle-higher-income, middle-lower-income and low-income regions) in Chengdu were purposefully selected for the study.

Eligibility criteria for participation included: (a) female adolescents aged 15–18 years; (b) residing in communities covered by the selected health centres; (c) no prior receipt of any HPV vaccine; (d) no known history of vaccine-related allergies; and (e) provision of caregiver consent to participate in the study.

### Randomisation and procedures

Caregivers of eligible girls were invited by telephone calls following simple randomisation of the name lists recorded in the community administrative registration system until we reached the target sample size from each community. Participants with caregivers were invited to the vaccination clinics and randomly assigned to the two arms using the envelope concealment method.

### Intervention

A total of 321 participants were recruited from the four sites, with 160 allocated to the standard-of-care group and 161 to the pay-it-forward group. Participants in the standard-of-care group received a health education leaflet about the HPV vaccine commonly used in clinics. They needed to self-pay the vaccine’s cost at the market price if they decided to vaccinate. Participants in the pay-it-forward group were informed that previous participants had donated 329 RMB (47.7 USD), equivalent to the cost of one dose of the domestic 2v-HPV vaccine, to support their vaccination. Additionally, they received postcard messages co-designed by college students tackling knowledge gaps (online supplemental figure S1). The health education materials focus on: (a) the effectiveness of various HPV vaccines in preventing cervical cancer; (b) the importance of early vaccination for eligible girls; and (c) parental perspectives on the age at which their daughters begin sexual activity. Following vaccination, participants were given the opportunity to donate and/or write postcard messages, fostering encouragement and community solidarity among peers.

### Sample size

The sample size calculation for the original trial was reported in a previous article.^16^ While the primary trial was powered for 321 participants, no formal sample size was calculated for the mediation analysis, which may have affected the statistical power of this analysis.

### Outcome measures

The primary outcome was the receipt of the first dose of HPV vaccine following the intervention within 3 months. This was ascertained by clinical vaccination records obtained from the community health service centres.

### Putative mediators

Data on mediator variables were collected through a self-administered online questionnaire after the intervention. During the study period, only the domestic 2v-HPV vaccine (Cecolin) was consistently available at the four community health service centres. Based on previous literature, we hypothesised that vaccine confidence^17^ and vaccine delay intention^18 19^ were potential mediators.

Vaccine confidence was measured using the vaccine confidence index (VCI),^20^ which centres on perception of vaccine safety, effectivenes and importance. Its concise format enhances participant adherence. The VCI has demonstrated strong applicability in prior research on influenza vaccine confidence conducted in China.^21 22^ Each participant indicated their level of agreement with three statements related to the vaccine: ‘Overall, I believe the 2v-HPV vaccine is important’; ‘Overall, I believe the 2v-HPV vaccine is safe’; and ‘Overall, I believe the 2v-HPV vaccine is effective’.^17 23^ Responses to these statements were recategorised into binary variables: ‘agree’ (including ‘strongly agree’ and ‘agree’) and ‘disagree’ (combining ‘strongly disagree’ and ‘disagree’).

Given the high willingness of Chinese parents to vaccinate and their widespread preference for the 9v-HPV vaccine—despite its limited supply—this study explored whether they would proceed with vaccination without delay if the preferred vaccine were easily accessible. To capture the specific Chinese context, a self-designed, scenario-based measure was used to assess intention of the participants to delay vaccination. Caregivers were asked what they would do if the 9v-HPV or 4v-HPV vaccines were not available at their community health service centre. Participants opting for the available 2v-HPV vaccine (whether domestic or imported) were considered as not intending to delay vaccination. Conversely, those who chose to postpone vaccination temporarily and wait until the 4v-HPV or 9v-HPV vaccines became available were considered as having vaccine delay intention.

### Confounders

Because participants were randomly assigned, we assumed there were minimal confounding factors affecting both the intervention-mediator and intervention-outcome models. A directed acyclic graph (DAG)^24^ was constructed to detect possible pretreatment confounding variables between each mediator-outcome relationship, as depicted in online supplemental figures S2 and S3. The DAGs implied that the following factors needed to be adjusted for potential confounding: sex of guardian, annual household income, education level, marital status, daughter’s age, guardian’s age and awareness of 2v-HPV prior to study enrolment.

### Statistical analysis

To explore how the pay-it-forward intervention might have worked in sub-populations with diverse sociodemographic backgrounds, we stratified participants into urban and suburban areas according to where the study took place geographically. We used multivariable logistic regression models to examine the associations of interventions (standard-of-care and pay-it-forward groups) with vaccine uptake, vaccine confidence, and vaccine delay intention, adjusting for sex of guardian, annual household income, education level, marital status, daughter’s age, and awareness of 2v-HPV.

**Figure 1:**
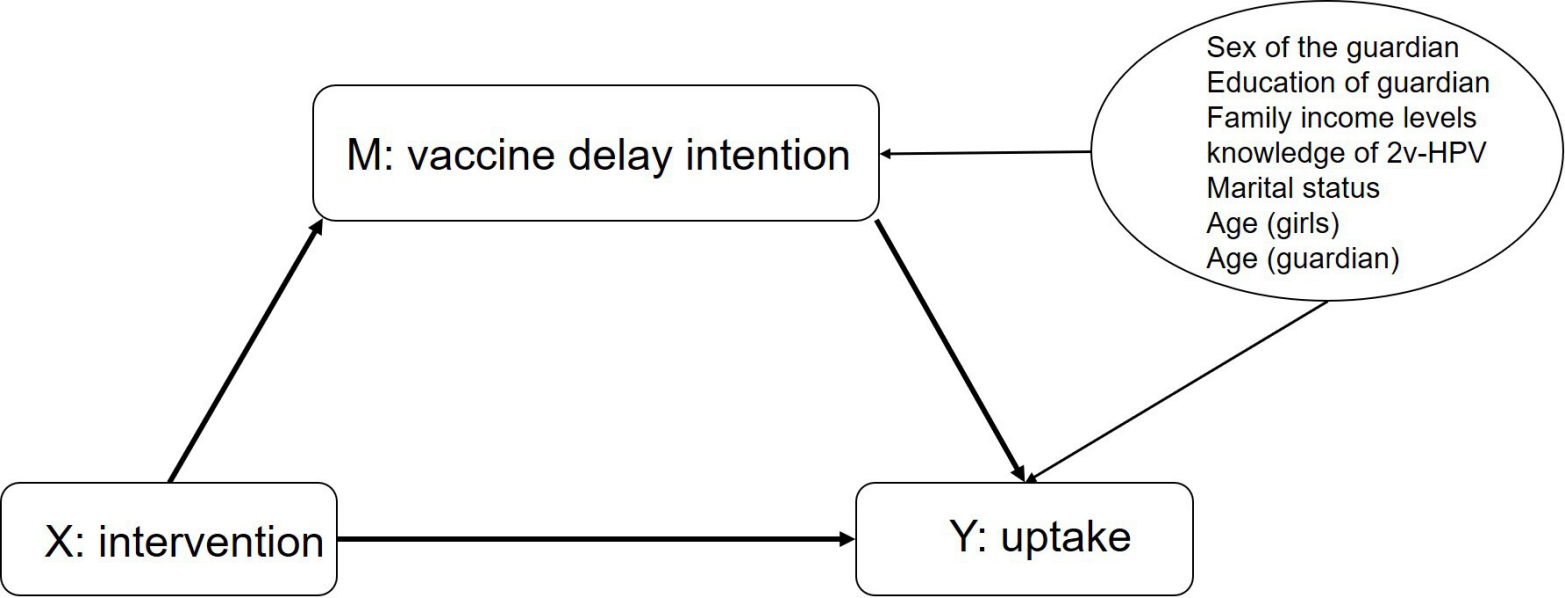
Directed acyclic graph of hypothesised mechanisms in single mediator model.

For mediation analyses, we explored the multiple mediator models, including both vaccine confidence and vaccine delay intention. Given the potential correlation between the two, vaccine confidence (as an alternative mediator) could act as a post-randomisation confounder for the relationship between the primary mediator (vaccine delay intention) and the outcome (HPV uptake).^25^ However, our analysis found no significant association between vaccine confidence and vaccine delay intention (online supplemental table S1), indicating limited interaction between the two mediators. Moreover, the multiple mediator model did not yield meaningful mediation effects. Additionally, the single mediator models revealed only small proportions of mediation effects. Therefore, we aimed to determine a single mediating mechanism, achieved by constructing a single mediator model to evaluate the impact of changes in vaccine confidence and vaccine delay intention on HPV uptake. A single mediator model was constructed for every mediator (including the three dimensions of vaccine confidence—safety, effectiveness, and importance—as well as vaccine delay intention) and the uptake outcome (figure 1).

Causal mediation analysis is usually preferred because it can accommodate more realistic settings, such as non-linear relations.^13 14 26^ In our study, each single mediation model employed a model-based^12^ causal mediation analysis. This method allowed us to estimate the direct and indirect effects by fitting two statistical models: the mediation model and the outcome model. The mediation model was built with intervention assignment as the independent variable and the mediator as the dependent variable. Conversely, in the outcome model, the mediator serves as an independent variable, and the outcome is the dependent variable, with treatment allocation and a set of potential confounding factors for the mediator-outcome relationship as covariates. Given the binary nature of our dependent variables, we used generalised linear regression models (logit) for all analyses. As recommended, we incorporated the interaction between intervention and mediator in the models, as not doing so might lead to biased estimates and reduce the statistical power to detect indirect effects.^13^ We used the ‘mediation’ package^27^ for conducting mediation analysis and the ‘mediate’ function was used to compute the intervention-mediator effect (Path a), the mediator-outcome effect (Path b), average total effect (ATE), average causal mediation effect (ACME) and average direct effect. The proportion mediated was calculated by dividing the indirect effect by the total effect, indicating the extent to which the mediator accounts for the overall effect. We used 2000 non-parametric bootstrap simulations to generate 95% CIs.

We performed sensitivity analyses to evaluate the robustness of the ACME to bias from residual confounding. Because both the outcome and mediators were binary variables, the ‘medsens’ function in the ‘mediation’ package was not appropriate for sensitivity analysis.^27^ Instead, we used the ‘sensmediation’ package^28^ for conducting sensitivity analysis on the mediation effects. This package is suitable for binary variables and yields mediation analysis results akin to those from the ‘mediation’ package.^28^ The ‘sensmediation’ package estimates the level of unmeasured confounding that would reduce the ACME to zero. This is done by examining the correlation (rho) between the residuals of the mediator and outcome models. A non-zero rho indicates the presence of unmeasured confounding in the mediator-outcome relationship. By calculating the ACME (point estimates and 95% CIs) across a range of rho values (from − 1 to +1), we can evaluate how strong the unmeasured confounding would need to be to nullify the observed mediation effect. The higher the rho value required to reduce the ACME to zero, the more robust the mediation effect is to unmeasured confounding. The sensitivity parameter rho was interpreted using Cohen’s benchmarks: small (≥0.10), medium (≥0.30) and large (≥0.50).^29^

The post hoc analysis was conducted to explore factors affecting HPV vaccine uptake. χ^2^ tests were used to compare preintervention knowledge of HPV and HPV vaccines between caregivers of vaccinated and unvaccinated girls, stratified by urban and suburban populations. Statistical significance was assessed using a two-sided significance level of 0.05, with all analyses conducted in R 4.4.0.

## Results

A total of 321 participants were enrolled in the study. Baseline characteristics of participants were well balanced between the two groups, except in suburban areas where the pay-it-forward group had a slightly higher proportion of male caregivers compared with the standard-of-care group (table 1).

**Table 1:** Baseline characteristics of participants in urban and suburban areas

	Suburban areas			Urban areas		
	Standard-of-care (n=76)	Pay-it-forward (n=79)	P	Standard-of-care (n=84)	Pay-it-forward (n=82)	P
Age (girls)—years	16.6±1.0	16.7±0.9	0.423	16.7±0.9	16.5±1.1	0.150
Age (caregivers)—years	45.5±9.8	42.9±8.0	0.070	43.3±6.9	43.2±8.4	0.949
Sex of the caregiver			**0.005**			0.601
Male	9 (11.8)	24 (30.4)		17 (20.2)	14 (17.1)	
Female	67 (88.2)	55 (69.6)		67 (79.8)	68 (82.9)	
Marital status of the caregivers			0.909			0.132
Unmarried	10 (13.1)	12 (15.2)		4 (4.8)	11 (13.4)	
Married	62 (81.6)	62 (78.5)		72 (85.7)	62 (75.6)	
Divorced or widowed	4 (5.3)	5 (6.3)		8 (9.5)	9 (11.0)	
The education level of the caregiver			0.556			0.892
High school or less	66 (86.8)	71 (89.9)		48 (57.1)	46 (56.1)	
College or more	10 (13.2)	8 (10.1)		36 (42.9)	36 (43.9)	
Income*			0.219			0.323
≤11,611 USD/year	62 (81.6)	70 (88.6)		48 (57.1)	53 (64.6)	
> 11,611 USD/year	14 (18.4)	9 (11.4)		36 (42.9)	29 (35.4)	
Have you ever heard of HPV?			0.590			>0.999
Yes	62 (81.6)	67 (84.8)		80 (95.2)	78 (95.1)	
No	14 (18.4)	12 (15.2)		4 (4.8)	4 (4.9)	
Have you heard of the HPV vaccine?			0.748			0.097
Yes	64 (84.2)	65 (82.3)		82 (97.6)	75 (91.5)	
No	12 (15.8)	14 (17.7)		2 (2.4)	7 (8.5)	
Do you know that the 2v-HPV vaccine has been marketed in China (including domestic and imported)?			0.744			0.758
Yes	55 (72.4)	59 (74.7)		76 (90.5)	73 (89.0)	
No	21 (27.6)	20 (25.3)		8 (9.5)	9 (11.0)	

Data are mean (SD) or n (%). 1USD=6.89 RMB.

Boldface indicates statistical significance (p<0.05).

* The average household income in rural Sichuan Province was around RMB 80,000 in 2021, according to Sichuan Statistical Yearbook 2022.^49^

HPV, human papillomavirus.

Online supplemental table S2 revealed significant differences between the pay-it-forward group and the standard-of-care group among urban participants. More urban participants of the pay-it-forward group were reported to receive the HPV vaccine (adjusted odds ratio (aOR)=3.4, 95% CI 1.5 to 7.9) and exhibited higher levels of perceived importance (aOR=6.5, 95% CI 1.4 to 29.5), safety (aOR=4.2, 95% CI 1.0 to 16.9) and efficacy (aOR=4.8, 95% CI 1.2 to 19.0) regarding the 2v-HPV vaccine. In addition, the pay-it-forward group showed a lower proportion of vaccine delay intention (aOR=0.4, 95%CI 0.2 to 0.8).

For suburban participants, those in the pay-it-forward group were more likely to receive the HPV vaccine compared with the standard-of-care group (aOR=2.6, 95% CI 1.1 to 6.1). However, no statistically significant differences were observed between the two groups in terms of vaccine confidence or intention to delay vaccination (p values>0.05).

As shown in table 2, among all participants, vaccine delay intention mediated 28% (95% CI 0.01 to 0.55) of the ATE on vaccination. Among urban participants, vaccine delay intention has a significant indirect impact on HPV vaccination (0.08; 95% CI 0.01 to 0.15), accounting for an average proportion of mediating effects of 39% (95% CI 0.06 to 0.95). This finding suggests that reducing vaccine delay intention accounted for about 39% of the intervention’s effect on HPV vaccination among urban participants and 28% of the effect in the overall population.

**Table 2:** Total, indirect and direct effect estimates of vaccine confidence and vaccine delay intention as mediator

		Vaccine delay intention Estimate (95% CI)	Vaccine confidence Estimate (95% CI)		
			Importance	Safety	Efficacy
Urban participants	Path a*	**0.93** (0.29, 1.58)	**2.50** (0.83, 5.42)	**1.38** (0.17, 2.89)	**1.18** (0.07, 2.19)
	Path b†	**2.43** (1.05, 4.07)	0.48 (−1.45, 3.50)	0.65 (−1.24, 3.66)	1.149 (−0.80, 4.19)
	ACME	**0.08** (0.01, 0.15)	0.03 (−0.02, 0.05)	0.023 (−0.01, 0.05)	0.02 (−0.01, 0.05)
	ADE	**0.13** (0.01, 0.25)	**0.183** (0.07, 0.32)	**0.18** (0.06, 0.31)	**0.17** (0.06, 0.31)
	TE	**0.21** (0.07, 0.35)	**0.211** (0.08, 0.34)	**0.20** (0.08, 0.33)	**0.20** (0.08, 0.33)
	Prop. mediated	**0.39** (0.06, 0.95)	0.133 (−0.08, 0.29)	0.11 (−0.02, 0.28)	0.12 (−0.03, 0.32)
Total sample	Path a*	**0.60** (0.15, 1.06)	**0.89** (0.05, 1.80)	**1.15** (0.30, 2.11)	**0.78** (0.04, 1.57)
	Path b†	**2.12** (1.20, 3.15)	−0.01 (−1.27, 1.54)	−0.22 (−1.37, 1.13)	−0.23 (−1.32, 1.00)
	ACME	**0.05** (0.01, 0.09)	−0.01 (−0.03, 0.01)	0.01 (−0.02, 0.03)	0.01 (−0.01, 0.02)
	ADE	**0.13** (0.04, 0.22)	**0.18** (0.09, 0.27)	**0.166** (0.07, 0.26)	**0.17** (0.08, 0.26)
	TE	**0.18** (0.01, 0.27)	**0.17** (0.08, 0.26)	**0.174** (0.08, 0.27)	**0.17** (0.08, 0.26)
	Prop. mediated	**0.28** (0.01, 0.55)	−0.04 (−0.19, 0.08)	0.042 (−0.10, 0.18)	0.03 (−0.07, 0.15)

Boldface indicates statistical significance (p<0.05).

* Path a is intervention-mediator effect.

† Path b is mediator-outcome effect.

ACME, average causal mediating effect; ADE, average direct effect; Prop. Mediated, average proportion of mediating effects; TE, total effect.

In both total participants and those from urban areas, confidence in the safety, effectiveness and importance of the bivalent vaccine did not appear to clarify the impact of the pay-it-forward intervention on HPV vaccination behaviour. All intervention-mediator (ie, Path a) estimates were significant, whereas all mediator-outcome (ie, Path b) estimates were not significant.

Sensitivity analysis indicated that these effects were likely robust against residual confounding. Specifically, the critical rho value at which the ACME would diminish to zero is 0.7 for urban participants and 0.6 for the total sample (online supplemental figures S4 and S5).

Since there were no significant differences in putative mediators between the pay-it-forward group and the standard-of-care group among suburban residents, additional analyses to assess causal mediation effects were not conducted.

### Post hoc analysis

In suburban populations, the pay-it-forward intervention effectively increased HPV vaccination rates. However, there was no significant difference in vaccine delay intention and vaccine confidence between the pay-it-forward and the standard-of-care group. The findings revealed that, among suburban participants, caregivers whose daughters were vaccinated through the programme had lower baseline awareness of HPV and HPV vaccines than those who chose not to vaccinate (table 3). Specifically, prior to joining the project, 41.5% of vaccinated suburban participants did not know that the 2v-HPV vaccine was accessible in China, whereas only 21.1% of unvaccinated suburban participants lacked this awareness. Among urban participants, there was no statistically significant difference in pre-intervention vaccine knowledge between caregivers of vaccinated and unvaccinated girls (p values>0.05).

**Table 3:** Comparison of pre-intervention vaccine awareness among guardians of vaccinated versus unvaccinated girls, stratified by urban and suburban areas

	Total (n=166)	Urban				Suburban		
		Unvaccinated (n=124)	Vaccinated (n=42)	P	Total (n=155)	Unvaccinated (n=114)	Vaccinated (n=41)	P
Have you ever heard of HPV?				0.692				**0.013**
Yes	158 (95.2)	119 (96.0)	39 (92.9)		129 (83.2)	100 (87.7)	29 (70.7)	
No	8 (4.8)	5 (4.0)	3 (7.1)		26 (16.8)	14 (12.3)	12 (29.3)	
Have you heard of the HPV vaccine?				0.335				**0.003**
Yes	157 (94.6)	119 (96.0)	38 (90.5)		129 (83.2)	101 (88.6)	28 (68.3)	
No	9 (5.4)	5 (4.0)	４(9.5)		26 (16.8)	13 (11.4)	13 (31.71)	
Do you know that the 2v-HPV vaccine has been marketed in China (including domestic and imported)?				0.961				**0.011**
Yes	149 (89.8)	112 (90.3)	37 (88.1)		114 (73.5)	90 (78.9)	24 (58.5)	
No	17 (10.2)	12 (9.7)	5 (11.9)		41 (26.5)	24 (21.1)	17 (41.5)	

Boldface indicates statistical significance (p<0.05).

HPV, human papillomavirus.

## Discussion

This is a rare study that assessed potential mediators of a community-engaged pay-it-forward intervention on HPV vaccination, specifically focusing on changes in vaccine confidence and vaccine delay intention. Causal mediation analyses revealed that in urban areas, the effect of the pay-it-forward intervention on HPV vaccination was largely attributed (39%) to a decrease in vaccine delay intention. No significant mediating effect was observed for vaccine confidence. Among suburban participants, no significant mediation effects were found. Interestingly, among vaccinated individuals in suburban areas, a higher proportion lacked prior awareness about HPV vaccines compared with those who were unvaccinated.

We found that vaccine delay intention may be an important mediator for the pay-it-forward behavioural intervention. Among urban populations, approximately 39% of the intervention’s effect on HPV vaccination behaviour was mediated through reduced vaccine delay intention. This suggests that addressing delay intentions—particularly the tendency to wait for the 9-valent vaccine—may be a key pathway through which behavioural interventions influence vaccination uptake. Interventions that enhance awareness of available vaccine options and encourage timely action within the optimal vaccination window may be especially effective in urban settings. Incorporating these elements into future health communication and service delivery strategies could help reduce vaccination delays and improve overall coverage.

However, we focused on vaccine type preference as a key reason for vaccine delay, while acknowledging that other important factors also influence vaccination decisions. Previous research indicated that vaccine delay is related to costs, lack of vaccine confidence, poor awareness of available vaccine products and associated benefits.^30^,^32^ The pay-it-forward intervention had several key components that might have addressed the above factors. First, the pay-it-forward incentive and reciprocal messages might cultivate a sense of social solidarity and contribute to community trust. Previous review of evidence suggested that these can help enhance confidence in services.^33^ Second, despite the high willingness to self-pay for the 9v-HPV vaccine,^6 10 34^ the structural barrier of insufficient supply of the 9v-HPV vaccine^35 36^ prevented many from timely uptake. Our intervention provided information about other alternative available options that might have helped parents with informed decision-making. Third, the educational component included postcard messages that informed parents about different protection effects by age of vaccination, the median age of sexual debut and the potential risk of exposure.^16^ This may help parents recognise disease susceptibility and the importance of timely vaccination.

Our data further found that among urban participants, the pay-it-forward intervention was significantly associated with enhanced vaccine confidence compared with standard-of-care practice. This is consistent with a previous analysis that identified pay-it-forward intervention’s association with increased vaccine confidence levels.^33^ This has important implications for addressing vaccine hesitancy which was considered one of the 10 global public health threats by the WHO in 2019.^37^ However, vaccine confidence did not seem to be a mediator of the association between pay-it-forward and HPV vaccine uptake in this analysis. This might be attributed to several factors, including the government’s introduction of a subsidisation policy for HPV vaccination targeting 13–14-year-old school girls starting in 2021,^38^ resulting in increased dissemination of vaccine information and the general public’s trust in government and healthcare. In addition, parents with high levels of vaccine confidence were more likely to join our trial. These factors likely resulted in a high baseline vaccine confidence level (>85%) among participants before the intervention, which potentially could have masked the impact of changes in vaccine confidence on vaccination rates, especially given the small sample size. Further research is needed to understand the role of vaccine confidence in the pay-it-forward intervention and vaccine uptake, and other potential pathways.

Another interesting finding in our study was that in suburban areas, the effectiveness of the pay-it-forward intervention fell short of expectations, but caregivers who vaccinated their daughters had lower pre-intervention awareness of the HPV vaccine compared with those who did not vaccinate their daughters. It appears that caregivers with limited initial knowledge may be motivated to vaccinate once they receive relevant information, whereas those with greater prior awareness might still decline vaccination due to other factors such as cost, vaccine accessibility, preference for certain vaccine types and prevailing social norms.^39^,^42^ This highlights that vaccination decisions are influenced by a range of factors. This was in line with findings from previous studies,^43 44^ suggesting that improving HPV awareness in low-resource areas might enhance vaccination uptake. Our interpretation is that factors such as inadequate health resources,^45^ and economic and educational barriers in suburban areas may restrict access to information and reduce initiation in seeking healthcare services.^46 47^ In our study, the educational component might help bridge knowledge and awareness gaps, and improve vaccine uptake among suburban participants. Among urban participants who have better awareness, they likely have more vaccine hesitancy and confidence issues due to misinformation.^48^ These findings suggest the necessity of tailoring intervention materials to local contexts.

However, the study has several limitations. First, because the RCT was not specifically designed for mediation analysis, the identification and measurement of mediators were predetermined and focused on a limited set of potential factors. Future qualitative research with trial participants could be conducted to understand potential impact of different components of the pay-it-forward strategy. Second, the sample size was not determined based on the need for a causal mediation analysis, limiting the power of our analyses. But our analyses identified potential associations and provided a direction for future examination on potential mediation effects of vaccine confidence on uptake behaviours. We recommend that future vaccination behavioural trial designs could factor in vaccine confidence as a potential mediator to further validate its role through collecting more robust trial data. Third, there is a possibility of unmeasured confounders mixing with residual confounders. However, we conducted sensitivity analyses to ensure the reliability and robustness of these findings. Finally, while the measures of vaccine confidence and delay intention were brief and feasible, they were somewhat narrow. Vaccine confidence was assessed solely using the VCI, and delay intention was defined mainly by vaccine type preference, potentially overlooking key factors such as risk perception, trust in healthcare, financial constraints and social influences—limiting the ability to fully capture the complexity of vaccine decision-making.

## Conclusion

Our study indicates that in affluent urban areas, vaccine delay intention potentially mediates the influence of pay-it-forward interventions on vaccination uptake. A pay-it-forward strategy could improve vaccine confidence effectively, but vaccine confidence did not show a significant mediating effect. In remote areas with limited access to information, disseminating vaccine-related information could be a promising strategy to boost vaccination rates. There is a necessity to investigate more effective intervention approaches suitable for remote areas and to refine the information provided through pay-it-forward initiatives tailored to this population.

## Supplementary material

10.1136/bmjopen-2024-095248 online supplemental file 1

## Data Availability

Data are available upon reasonable request.

## References

[1] Singh D, Vignat J, Lorenzoni V (2023). Global estimates of incidence and mortality of cervical cancer in 2020: a baseline analysis of the WHO Global Cervical Cancer Elimination Initiative. Lancet Glob Health.

[2] Walboomers JM, Jacobs MV, Manos MM (1999). Human papillomavirus is a necessary cause of invasive cervical cancer worldwide. J Pathol.

[3] Zou Z, Fairley CK, Ong JJ (2020). Domestic HPV vaccine price and economic returns for cervical cancer prevention in China: a cost-effectiveness analysis. Lancet Glob Health.

[4] Tan S, Wang S, Zou X (2024). Parental willingness of HPV vaccination in Mainland China: A meta-analysis. Hum Vaccin Immunother.

[5] Chen J, Zhang Z, Pan W (2017). Estimated Human Papillomavirus Vaccine Coverage Among Females 9-45 Years of Age. China CDC Wkly.

[6] Zhou F, Zhang W, Cai H (2021). Portrayals of 2v, 4v and 9vHPV vaccines on Chinese social media: a content analysis of hot posts on Sina Weibo. Hum Vaccin Immunother.

[7] Wang Q, Zhang W, Cai H (2020). Understanding the perceptions of Chinese women of the commercially available domestic and imported HPV vaccine: A semantic network analysis. Vaccine (Auckl).

[8] Wu D, Liu P, Song D (2023). Implementing the free HPV vaccination for adolescent girls aged below 14 in Shenzhen, Guangdong Province of China: experience, challenges, and lessons. Infect Dis Poverty.

[9] Chen L, Sun X, Luo J (2023). A Case-Control Study on Factors of HPV Vaccination for Mother and Daughter in China. Vaccines (Basel).

[10] Lin Y, Su Z, Chen F (2021). Chinese mothers’ intention to vaccinate daughters against human papillomavirus (HPV), and their vaccine preferences: a study in Fujian Province. Hum Vaccin Immunother.

[11] Li J, Li Y, Qi C (2025). Pay-it-forward strategy reduced hpv vaccine delay and increased uptake among catch-up age girls: a randomized clinical trial. Public and Global Health.

[12] Imai K, Keele L, Tingley D (2010). A general approach to causal mediation analysis. Psychol Methods.

[13] VanderWeele T (2015). Explanation in causal inference: methods for mediation and interaction.

[14] Rijnhart JJM, Valente MJ, MacKinnon DP (2021). The use of traditional and causal estimators for mediation models with a binary outcome and exposure-mediator interaction. *Struct Equ Modeling*.

[15] Lee H, Cashin AG, Lamb SE (2021). A Guideline for Reporting Mediation Analyses of Randomized Trials and Observational Studies: The AGReMA Statement. JAMA.

[16] Li Y, Qin C, Qiu S (2023). The effectiveness of pay-it-forward in addressing HPV vaccine delay and increasing uptake among 15–18-year-old adolescent girls compared to user-paid vaccination: a study protocol for a two-arm randomized controlled trial in China. BMC Public Health.

[17] de Figueiredo A, Simas C, Karafillakis E (2020). Mapping global trends in vaccine confidence and investigating barriers to vaccine uptake: a large-scale retrospective temporal modelling study. Lancet.

[18] Yim VW-C, Wang Q, Li Y (2024). Between now and later: a mixed methods study of HPV vaccination delay among Chinese caregivers in urban Chengdu, China. BMC Public Health.

[19] Wei Z, Liu Y, Zhang L (2023). Stages of HPV Vaccine Hesitancy Among Guardians of Female Secondary School Students in China. J Adolesc Health.

[20] Larson HJ, Schulz WS, Tucker JD (2015). Measuring vaccine confidence: introducing a global vaccine confidence index. PLoS Curr.

[21] Wei Z, Sun X, Yang Y (2021). Seasonal influenza vaccine hesitancy profiles and determinants among Chinese children’s guardians and the elderly. Expert Rev Vaccines.

[22] Wu D, Jin C, Bessame K (2022). Effectiveness of a pay-it-forward intervention compared with user-paid vaccination to improve influenza vaccine uptake and community engagement among children and older adults in China: a quasi-experimental pragmatic trial. Lancet Infect Dis.

[23] Larson HJ, de Figueiredo A, Xiahong Z (2016). The State of Vaccine Confidence 2016: Global Insights Through a 67-Country Survey. EBioMedicine.

[24] Textor J, van der Zander B, Gilthorpe MS (2016). Robust causal inference using directed acyclic graphs: the R package “dagitty”. Int J Epidemiol.

[25] VanderWeele TJ, Vansteelandt S (2014). Mediation Analysis with Multiple Mediators. Epidemiol Methods.

[26] Cashin AG, McAuley JH, VanderWeele TJ (2023). Understanding how health interventions or exposures produce their effects using mediation analysis. BMJ.

[27] Tingley D, Yamamoto T, Hirose K (2014). mediation: R Package for Causal Mediation Analysis. J Stat Softw.

[28] Lindmark A, de Luna X, Eriksson M (2018). Sensitivity analysis for unobserved confounding of direct and indirect effects using uncertainty intervals. Stat Med.

[29] Cox MG, Kisbu-Sakarya Y, Miočević M (2013). Sensitivity plots for confounder bias in the single mediator model. Eval Rev.

[30] Sonawane K, Zhu Y, Montealegre JR (2020). Parental intent to initiate and complete the human papillomavirus vaccine series in the USA: a nationwide, cross-sectional survey. Lancet Public Health.

[31] Zhao F, Qiao Y (2019). Cervical cancer prevention in China: a key to cancer control. Lancet.

[32] Siu JY-M, Fung TKF, Leung LH-M (2019). Social and cultural construction processes involved in HPV vaccine hesitancy among Chinese women: a qualitative study. Int J Equity Health.

[33] Jiang W, Lu C, Yan X (2024). Vaccine confidence mediates the association between a pro-social pay-it-forward intervention and improved influenza vaccine uptake in China: A mediation analysis. Vaccine (Auckl).

[34] Shao X, Lu X, Zhou W (2023). HPV Vaccination Behavior, Vaccine Preference, and Health Beliefs in Chinese Female Health Care Workers: A Nationwide Cross-Sectional Study. Vaccines (Basel).

[35] You T, Zhao X, Hu S (2023). Optimal allocation strategies for HPV vaccination introduction and expansion in China accommodated to different supply and dose schedule scenarios: A modelling study. EClinicalMedicine.

[36] Wang X, Pan J, Yan B (2024). Inequities in human papillomavirus vaccination among children aged 9-14 years old under constrained vaccine supply in China. Int J Equity Health.

[37] World Health Organization (2019). Ten threats to global health in 2019.

[38] The People’s Government of Chengdu Municipality (2021). Chengdu will universally vaccinate schoolgirls aged 13-14 against HPV.

[39] Lin Z, Chen S, Su L (2024). Influences of HPV disease perceptions, vaccine accessibility, and information exposure on social media on HPV vaccination uptake among 11,678 mothers with daughters aged 9-17 years in China: a cross-sectional study. BMC Med.

[40] Vu M, Bednarczyk RA, Escoffery C (2022). U.S. Vietnamese parents’ HPV vaccine decision-making for their adolescents: an exploration of practice-, provider-, and patient-level influences. J Behav Med.

[41] Anandarajah A, Shato T, Humble S (2024). The association of caregiver attitudes, information sources, and trust with HPV vaccine initiation among adolescents. Hum Vaccin Immunother.

[42] Morgan JC, Badlis S, Head KJ (2025). That Parents Find Most Concerning: Insights From a Cross-Sectional Survey and Content Analysis. J Med Internet Res.

[43] Gong X, Xu J, He Y (2024). Socioeconomic inequalities in human papillomavirus knowledge and vaccine uptake: evidence from a cross-sectional study in China. Front Public Health.

[44] Mohammed KA, Subramaniam DS, Geneus CJ (2018). Rural-urban differences in human papillomavirus knowledge and awareness among US adults. Prev Med.

[45] Han K, Hou Z, Tu S (2022). Investigate Non-EPI Vaccination Recommendation Practice from a Socio-Ecological Perspective: A Mixed-Methods Study in China. Vaccines (Basel).

[46] Jia C, Long Y, Luo X (2022). Inverted U-shaped relationship between education and family health: The urban-rural gap in Chinese dual society. Front Public Health.

[47] Stephens ES, Dema E, McGee-Avila JK (2023). Human Papillomavirus Awareness by Educational Level and by Race and Ethnicity. JAMA Netw Open.

[48] Wang D, Wu J, Du J (2022). Acceptability of and barriers to human papillomavirus vaccination in China: A systematic review of the Chinese and English scientific literature. European J Cancer Care.

[49] Sichuan Province Bureau of Statistics (2022). Sichuan Statistical Yearbook 2022: Sichuan Province Bureau of Statistics.

