# Supplementary material for: Causal mediation analysis of a randomised controlled trial in China: evaluating whether the pay-it-forward strategy increases HPV vaccine uptake by reducing vaccine delay intention and increasing vaccine confidence
Source: BMJ Open. 2025 Sep 25;15(9):e095248. doi: 10.1136/bmjopen-2024-095248 (PMC12481287; doi:10.1136/bmjopen-2024-095248)
Supplement: online supplemental file 1 [file bmjopen-15-9-s001.docx]

**Supplementary**

**Postcard messages co-designed by college students**

| 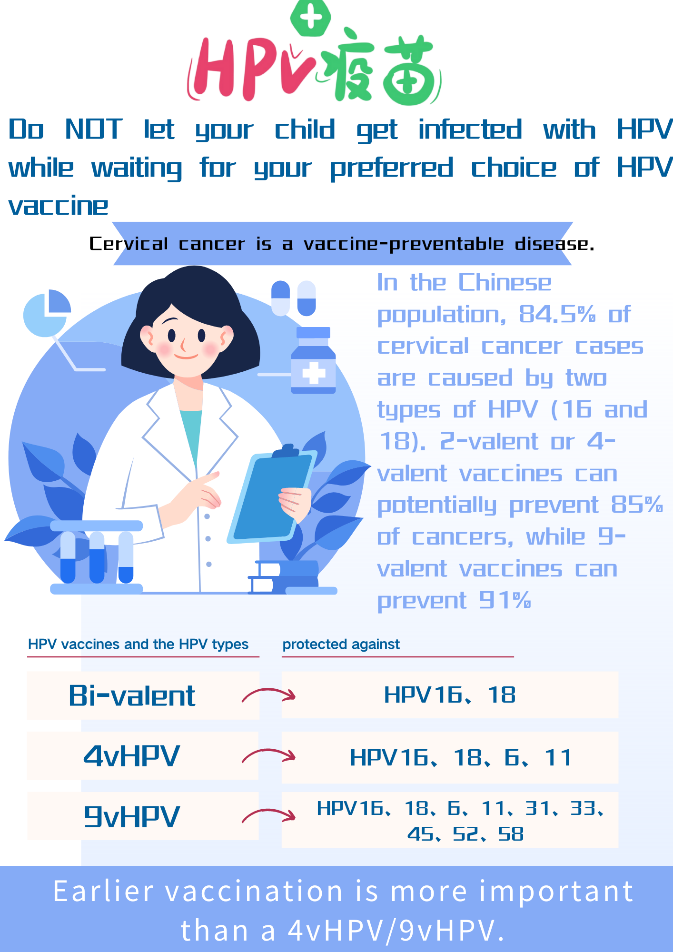 |
| --- |
| Reference: Chen W, Zhang X, Molijn A, Jenkins D, Shi JF, Quint W, Schmidt JE, Wang P, Liu YL, Li LK, Shi H, Liu JH, Xie X, Niyazi M, Yang P, Wei LH, Li LY, Li J, Liu JF, Zhou Q, Hong Y, Li L, Li Q, Zhou HL, Bian ML, Chen J, Qiao YL, Smith JS. Human papillomavirus type-distribution in cervical cancer in China: the importance of HPV 16 and 18. Cancer Causes Control. 2009 Nov;20(9):1705-13. |
| 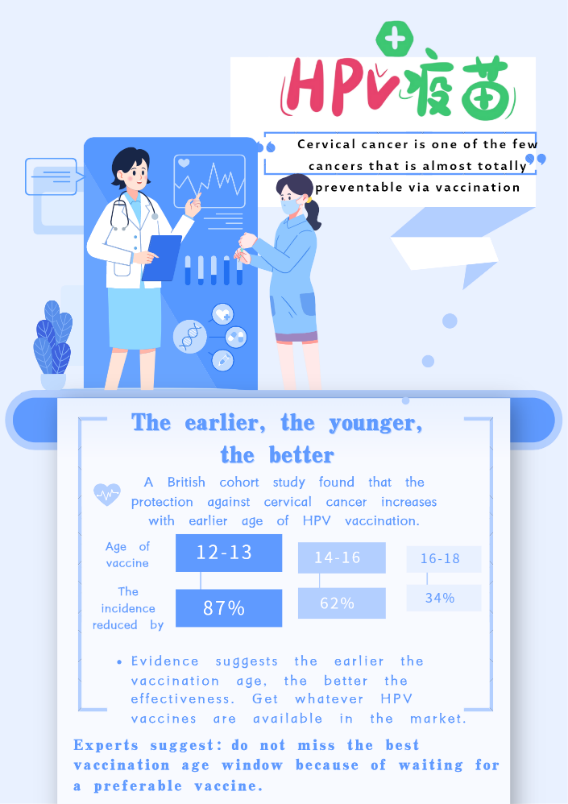 |
| Reference: Falcaro M, Castañon A, Ndlela B, Checchi M, Soldan K, Lopez-Bernal J, et al. The effects of the national HPV vaccination programme in England, UK, on cervical cancer and grade 3 cervical intraepithelial neoplasia incidence: a register-based observational study. Lancet. 2021;398(10316):2084–92. |
| 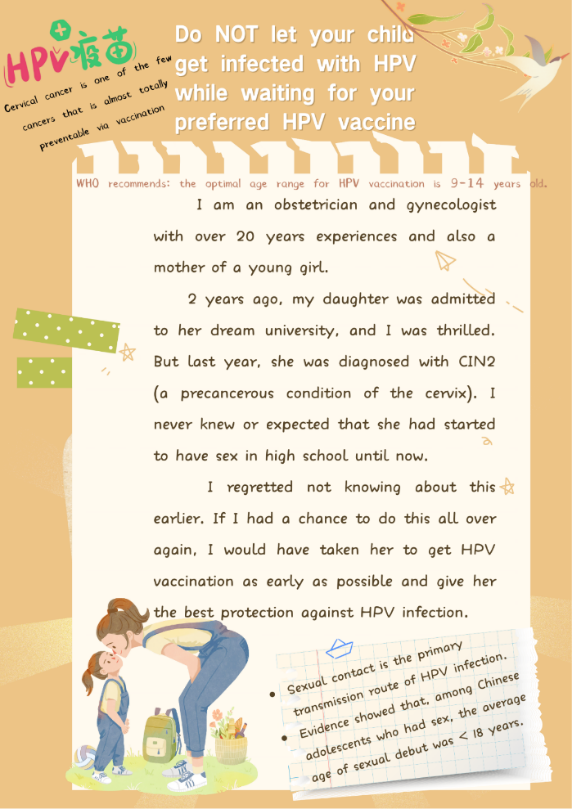 |
| Reference: Yang J, Shen M, Wang Z, et al. Prevalence and influencing factors of sexual behavior among university freshmen. Chinese Journal Public Health 2021; 37(03): 431-4. |

Figure S1 Community engaged postcards

**Directed acyclic graph**


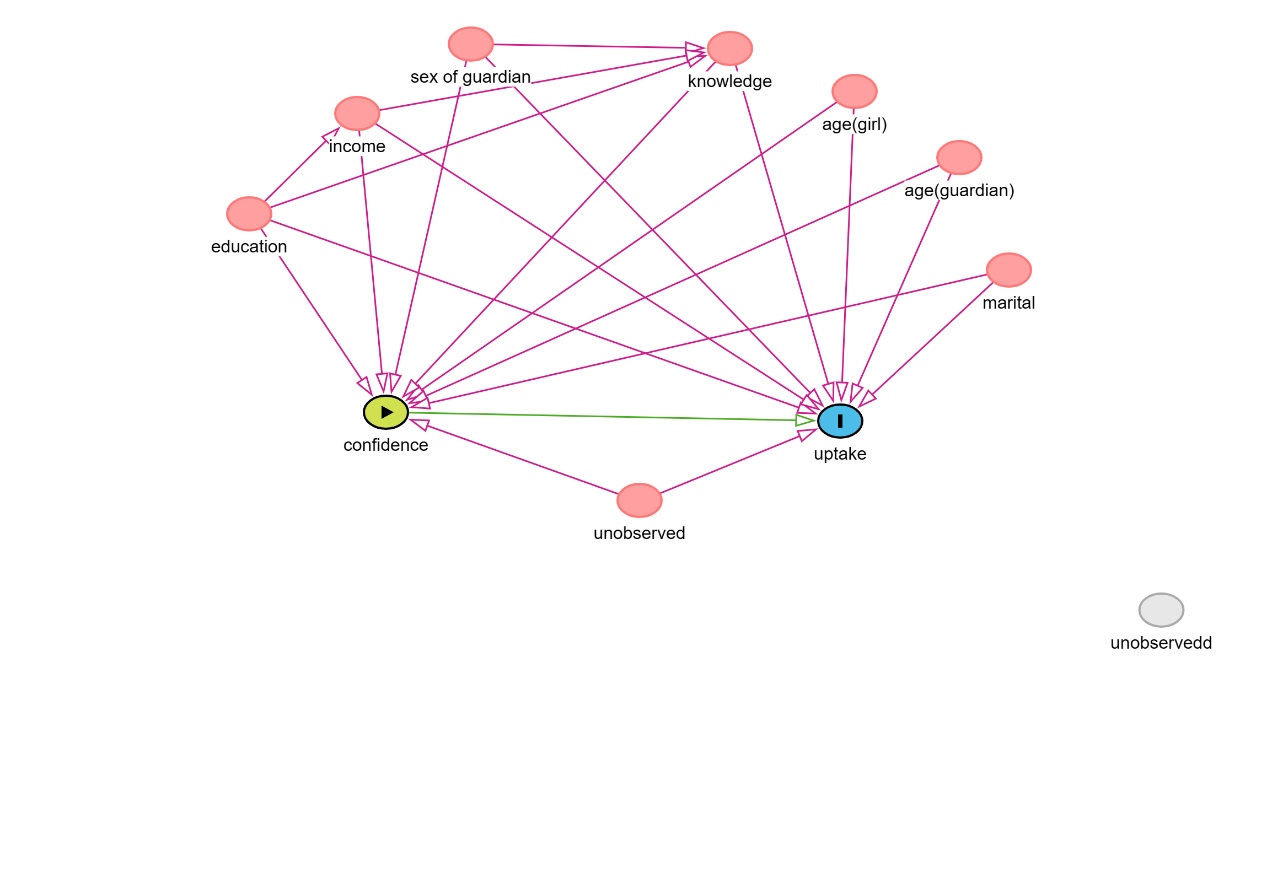


Figure S2 Directed acyclic graph of hypothesized mechanisms in the mediator-outcome relationship when vaccine confidence is used as the mediating variable.


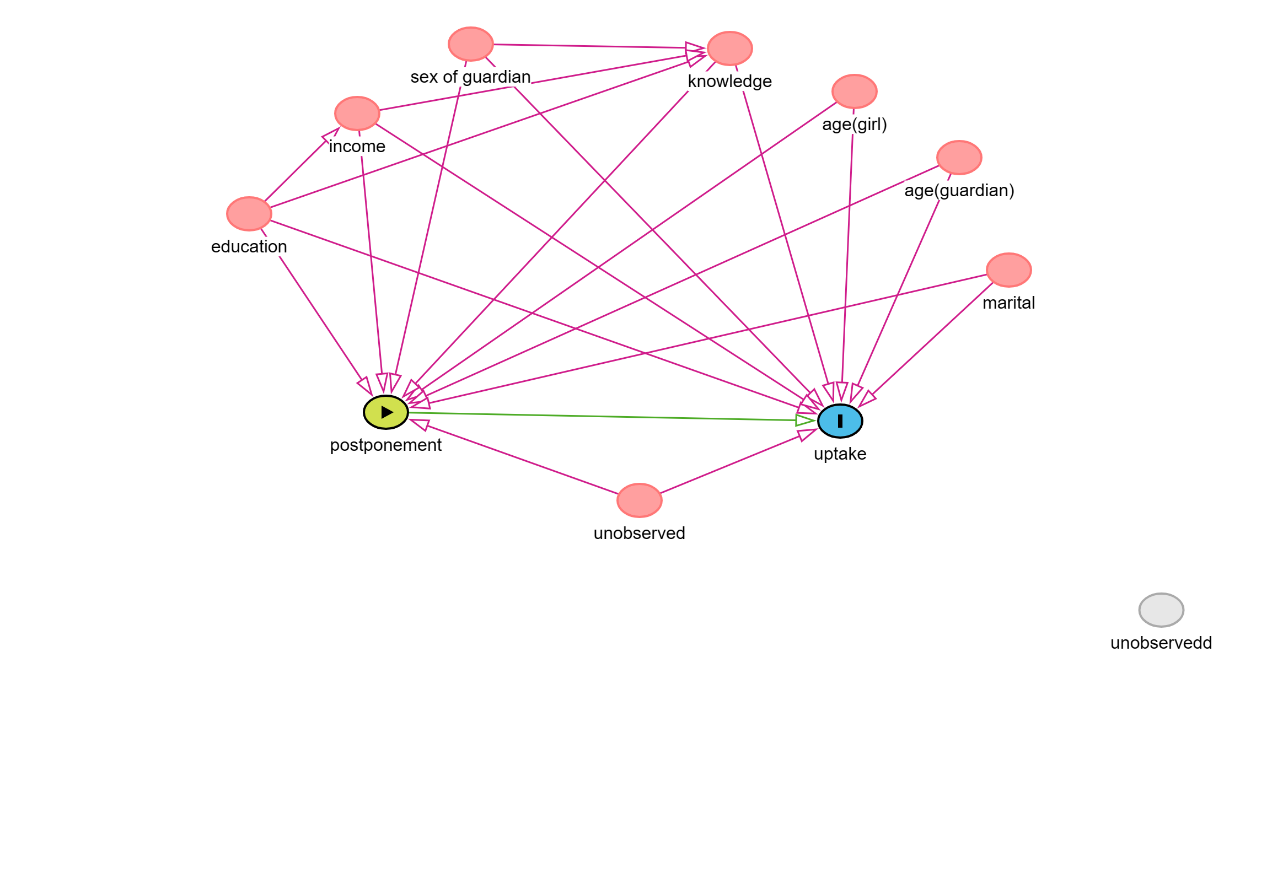


Figure S3 Directed acyclic graph of hypothesized mechanisms in the mediator-outcome relationship when willingness to postpone vaccine is used as the mediating variable.

**Table S1** Association Between Vaccine Confidence and Vaccine Delay Intention. (Chi-square Test)

|  | Urban (N=166) | | |  | Total (N=321) | | |
| --- | --- | --- | --- | --- | --- | --- | --- |
|  | Vaccine delay intention | No vaccine delay intention | P |  | Vaccine delay intention | No vaccine delay intention | P |
| **Confidence in importance** |  |  | 0.515* |  |  |  | 0.190 |
| No | 9(8.7) | 3(4.8) |  |  | 19(9.7) | 7(5.6) |  |
| Yes | 94(91.3) | 60(95.2) |  |  | 177(90.3) | 118(94.4) |  |
| **Confidence in safety** |  |  | 0.857 |  |  |  | 0.532 |
| No | 9(8.7) | 5(7.9) |  |  | 18(9.2) | 9(7.2) |  |
| Yes | 94(91.3) | 58(92.1) |  |  | 178(90.8) | 116(92.8) |  |
| **Confidence in effectiveness** |  |  | 0.969 |  |  |  | 0.955 |
| No | 10(9.7) | 6(9.5) |  |  | 20(10.2) | 13(10.4) |  |
| Yes | 93(90.3) | 57(90.5) |  |  | 176(89.8) | 112(89.6) |  |

***** P value from chi-square test with Yates’ continuity correction

Table S2 Compare vaccine uptake, confidence, and vaccine delay intention between two groups after receiving the intervention. ^a^

|  | Urban (N=166) | | |  |  | Suburban (N=155) | | |  |  | Total (N=321) | | |  |
| --- | --- | --- | --- | --- | --- | --- | --- | --- | --- | --- | --- | --- | --- | --- |
|  | Pay-it-forward  n (%) | Standard-of-care  n (%)  (reference) | aOR (95%CI) | P |  | Pay-it-forward  n (%) | Standard-of-care  n (%)  (reference) | aOR (95%CI) | P |  | Pay-it-forward  n (%) | Standard-of-care  n (%)  (reference) | aOR (95%CI) | P |
| **Uptake** | 29(35.4) | 13(15.5) | 3.4(1.5,7.9) | **0.004** |  | 26(32.9) | 15(19.7) | 2.6(1.1,6.1) | **0.024** |  | 55(34.2) | 28(17.5) | 2.6(1.5,4.6) | **0.007** |
| **Confidence in importance** | 81(98.8) | 73(86.9) | 6.5(1.4,29.5)^b^ | **0.015** |  | 72(91.1) | 69(90.3) | 1.0(0.3,3.5) | 0.982 |  | 153(95.0) | 142(88.7) | 2.5(1.0,6.3) | **0.049** |
| **Confidence in safety** | 79(96.3) | 73(86.9) | 4.2(1.0,16.9) | **0.045** |  | 75(94.9) | 67(88.2) | 3.6(0.9,14.5) | 0.073 |  | 154(95.6) | 140(87.5) | 3.6(1.4,9.1) | **0.008** |
| **Confidence in effectiveness** | 78(95.1) | 72(85.7) | 4.8(1.2,19.0) | **0.027** |  | 72(91.1) | 66(86.8) | 1.7(0.6,5.4) | 0.337 |  | 150(93.2) | 138(86.3) | 2.5(1.1,5.6) | **0.027** |
| **Vaccine delay intention** | 42(51.2) | 61(72.6) | 0.4(0.2,0.8) | **0.007** |  | 45(5.0) | 48(63.2) | 0.7(0.3,1.3) | 0.262 |  | 87(54.0) | 109(68.1) | 0.5(0.3,0.8) | **0.007** |

Note: Boldface indicates statistical significance (p<0.05).

a The multivariable logistic regression model adjusted for sex of guardian, annual household income, education level, marital status, daughter’s age, and awareness of 2-valent HPV. aOR=adjusted odds ratio.

b Adjusted odds ratios (aOR) with 95% confidence intervals were calculated using profile likelihood. The logistic regression analysis of the original data produced (aOR=12.1, 95%CI 1.5 to 100.6; P=0.021). Given the small sample size, a Firth logistic regression was conducted.

**Sensitivity analysis**

Sensitivity analysis was conducted only when vaccine delay intention served as a mediator. The other potential mediators did not demonstrate significant mediating effects, so no additional sensitivity analyses were carried out.

Sensitivity analysis was conducted to examine the unmeasured confounding factors. The curve shows the average causal mediation effect (NIE) as it varies with the sensitivity parameter rho, which represents the correlation between the residuals of the mediator and outcome models, with confidence bands shown in gray. Assuming no unmeasured confounding in the mediator-outcome relationship, the estimated average causal mediation effect is observed when rho is zero (dashed line). For the average causal mediation effect to be zero, the rho value is 0.7 for urban participants and 0.6 for the total participants.


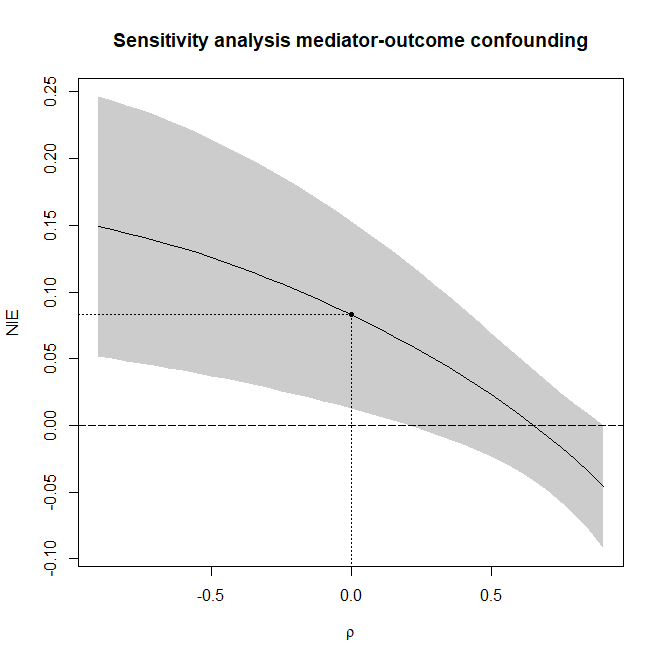


Figure S4 Sensitivity analysis of the average causal mediation effect when vaccine delay intention was used as a mediating variable in urban area.


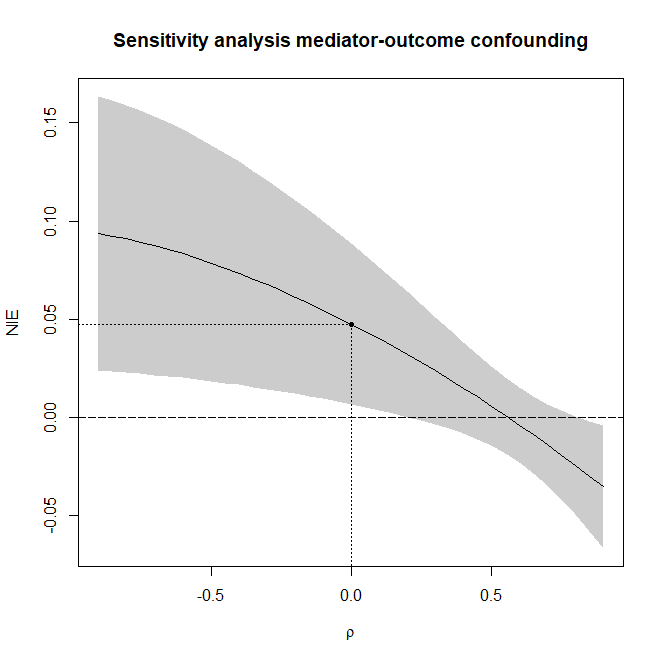


Figure S5 Sensitivity analysis of the average causal mediation effect when vaccine delay intention was used as a mediating variable in total participants.
