# Supplementary material for: Causal mediation analysis of a randomised controlled trial in China: evaluating whether the pay-it-forward strategy increases HPV vaccine uptake by reducing vaccine delay intention and increasing vaccine confidence
Source: BMJ Open. 2025 Sep 25;15(9):e095248. doi: 10.1136/bmjopen-2024-095248 (PMC12481287; doi:10.1136/bmjopen-2024-095248)
Supplement: Supplementary file 2 [file bmjopen-2024-095248.reviewer_comments.pdf]

PEER REVIEW HISTORY

BMJ Open publishes all reviews undertaken for accepted manuscripts. Reviewers are asked to complete a checklist review form and are provided with free text boxes to elaborate on their assessment. These free text comments are reproduced below.

ARTICLE DETAILS

Title (Provisional)

Causal Mediation Analysis of a Randomized Controlled Trial in China: Evaluating Whether the Pay-It-Forward Strategy Increases HPV Vaccine Uptake by Reducing Vaccine Delay Intention and Increasing Vaccine Confidence

Authors

Lu, Yajiao; Yang, Ying; Li, Yifan; Qin, Chuanyu; He, Yu; Gong, Wenfeng; Tang, Shenglan; Li, Jing; Wu, Dan

---

VERSION 1 - REVIEW

**Reviewer**	**1**
**Name**	**Shi, Baoyi**
**Affiliation**	**Columbia University Mailman School of Public Health**
**Date**	**28-Apr-2025**
**COI**	**None**

This paper reports mediation analyses from a randomized controlled trial among adolescent girls in China, examining whether vaccine confidence and vaccine delay intention mediate the effect of a pay-it-forward intervention on HPV vaccine uptake. The statistical methods are appropriate, and the paper is clearly written. However, the main finding—that reducing the cost of vaccination through the intervention decreases vaccine delay intention and thereby increases vaccine uptake—aligns with expectations and does not provide particularly novel insights into behavioral mechanisms beyond what is already well understood in the literature.

I have the following specific comments and suggestions:

1. The two mediators of interest—vaccine confidence and vaccine delay intention—are causally related to each other. Therefore, performing single mediation analysis with only one mediator at a time may not be valid, as the other mediator could act as a mediator–outcome confounder, leading to biased effect estimates. In this context, it is more appropriate to perform a joint mediation analysis that includes both mediators simultaneously. While single mediation analyses can be presented for descriptive purposes, the joint mediation analysis

should be considered the primary result. I recommend adding a joint mediation analysis and using it as the main finding.

2. Sex of the caregiver is so imbalanced across treatment groups. Could this suggest that randomization may not have been fully successful?

3. "Sensitivity analysis indicated that these effects were likely robust against residual confounding. Specifically, the critical rho value at which the average causal mediation effects (ACME) would diminish to zero is 0.7 for urban participants and 0.6 for the total sample (see Figure S4 and Figure S5 in the Supplementary). " Here, it would improve clarity to describe the definition of the rho value, how to interpret it, and why a value like 0.6 and 0.7 indicates robustness.

**Reviewer**	**2**
**Name**	**RIAD, Abanoub**
**Affiliation**	**Masaryk University Faculty of Medicine, Department of Public Health**
**Date**	**22-May-2025**
**COI**	**None**

Major Issues:

1. Sample Size Justification Absent

- The manuscript does not report a priori sample size calculation. Given the complexity of the behavioural intervention and the stratified mediation analysis, the achieved sample size (n = 224) may be underpowered to detect indirect effects with adequate precision.

- Recommendation: The authors should clarify whether the original RCT was powered to detect mediation effects. At minimum, a post hoc discussion of the sample size limitations should be added to the Study Limitations section.

2. Use of the Vaccine Confidence Index (VCI)

- While the VCI is widely used, applying it in isolation—especially with a relatively modest sample size—may not capture the multifaceted nature of vaccine confidence in the context of HPV vaccination in China.

- Recommendation: The authors should justify their reliance on VCI in this study and acknowledge its reductive nature in the limitations section. Additional indicators or triangulated measures would have strengthened this construct.

3. Inconsistencies Between Table 4 and Narrative Description

- Table 4 indicates that awareness of HPV infection, HPV vaccines, and vaccine types is higher among guardians of unvaccinated girls compared to those of vaccinated girls. However, the narrative in the Results and Discussion sections presents the opposite conclusion.

- Recommendation: This contradiction must be resolved. Either the table is mislabelled, the analysis misinterpreted, or the narrative needs to be corrected. This discrepancy directly affects the interpretation of vaccine-related knowledge as a barrier or enabler.

4. Interpretation of Mediation Effect Percentages

- The manuscript reports mediation percentages (e.g., 31.5%) but does not sufficiently explain how these were derived or what they imply in practical terms.

- Recommendation: The Discussion should offer a clearer interpretation of the mediation effect sizes, their public health relevance, and the assumptions under which they are valid.

5. Oversimplification of Vaccine Delay Intention

- The study frames delay intention as being primarily driven by the availability of HPV vaccine types, which is an important but limited factor.

- Recommendation: The authors should acknowledge that this is a narrow representation of delay intention. Other known contributors—such as caregiver risk perception, trust in healthcare providers, financial burden, and perceived social norms—should be discussed even if not measured.

Minor Issues:

1. Mislabelled Odds Ratios in Table 2

- Table 2 refers to “OR” but reports adjusted odds ratios. This mislabelling should be corrected to “aOR” to reflect the multivariable model outputs and maintain terminological accuracy.

VERSION 1 - AUTHOR RESPONSE

Response to reviewer 1

Comment 1

The two mediators of interest—vaccine confidence and vaccine delay intention—are causally related to each other. Therefore, performing single mediation analysis with only one mediator at a time may not be valid, as the other mediator could act as a mediator–outcome confounder, leading to biased effect estimates. In this context, it is more appropriate to perform a joint mediation analysis that includes both mediators simultaneously. While single mediation analyses can be presented for descriptive purposes, the joint mediation analysis should be considered the primary result. I recommend adding a joint mediation analysis and using it as the main finding.

Response 1

Thank you for your insightful comment. We agree that a joint mediation analysis is appropriate when the mediators are potentially related. In our study, we conducted a multiple mediator analysis including both vaccine confidence and vaccine delay intention to account for potential confounding between mediators. However, the analysis showed no significant association between vaccine confidence and vaccine delay intention (Table S1 in the Supplementary), suggesting minimal interdependence. Additionally, the multiple mediator model did not yield significant mediation effects for either pathway. Given these findings, we presented the single mediator models to descriptively explore the single mediation effects of vaccine confidence and vaccine delay intention. We have added the following clarification to the Methods section: "*For mediation analyses, we explored the multiple mediator models including both vaccine confidence and vaccine delay intention. Given the potential correlation between the two, vaccine confidence (as an alternative mediator) could act as a post-randomization confounder for the relationship between the primary mediator (vaccine delay intention) and the outcome (HPV uptake)*[25]*. However, our analysis found no significant association between vaccine confidence and vaccine delay intention (Table S1 in the Supplementary), indicating limited interaction between the two mediators. Moreover, the multiple mediator model did not yield meaningful mediation effects. Additionally, the single mediator models revealed only small proportions of mediation effects. Therefore, we aimed to determine a single mediating mechanism, achieved by constructing a single mediator model to evaluate the impact of changes in vaccine confidence and vaccine delay intention on HPV uptake.*" (In the "Statistical analysis" subsection of Methods, on page5, lines 2-12)

Added reference

VanderWeele TJ, Vansteelandt S. Mediation Analysis with Multiple Mediators. Epidemiol Methods. 2014;2(1):95-115. (See Reference)

Comment 2

Sex of the caregiver is so imbalanced across treatment groups. Could this suggest that randomization may not have been fully successful?

Response 2

Thank you for your insightful observation regarding the imbalance in caregiver sex across treatment groups. Participants at the four study sites were randomly assigned using a concealed envelope method administered by the same research team, which was designed to minimize allocation bias and generally resulted in balanced baseline characteristics.

However, we acknowledge that a higher proportion of male caregivers was observed in the rural intervention group after stratifying by urban and rural areas. This imbalance is likely attributable to chance, possibly compounded by the relatively small sample size within some strata, rather than a systematic failure of the randomization procedure.

To account for any potential confounding due to this imbalance, we adjusted for caregiver sex—as well as other key demographic variables such as age and education level—in all

subsequent regression models. We believe these steps adequately address the issue and support the validity of our study conclusions.

Comment 3

"Sensitivity analysis indicated that these effects were likely robust against residual confounding. Specifically, the critical rho value at which the average causal mediation effects (ACME) would diminish to zero is 0.7 for urban participants and 0.6 for the total sample (see Figure S4 and Figure S5 in the Supplementary). " Here, it would improve clarity to describe the definition of the rho value, how to interpret it, and why a value like 0.6 and 0.7 indicates robustness.

Response

Thank you for your valuable comment. We have added the sentence to clarify the definition and interpretation of the sensitivity parameter ρ (rho): *"The 'sensmediation' package estimates the level of unmeasured confounding that would reduce the ACME to zero. This is done by examining the correlation (rho) between the residuals of the mediator and outcome models. A nonzero rho indicates the presence of unmeasured confounding in the mediator-outcome relationship. By calculating the ACME (point estimates and 95% confidence intervals) across a range of rho values (from −1 to +1), we can evaluate how strong the unmeasured confounding would need to be to nullify the observed mediation effect. The higher the rho value required to reduce the ACME to zero, the more robust the mediation effect is to unmeasured confounding. The sensitivity parameter rho was interpreted using Cohen's benchmarks: small (⩾0.10), medium (⩾0.30), and large (⩾0.50).[29]* (In the "Statistical analysis" subsection of Methods, on page 5 (lines 32–42) and page 6 (lines 1-3))

Added reference

Cox MG, Kisbu-Sakarya Y, Miočević M, MacKinnon DP. Sensitivity plots for confounder bias in the single mediator model. Eval Rev. 2013;37(5):405-31. (See Reference)

Response to reviewer 2

Comment 1: Sample Size Justification Absent.

-The manuscript does not report a priori sample size calculation. Given the complexity of the behavioural intervention and the stratified mediation analysis, the achieved sample size (n = 224) may be underpowered to detect indirect effects with adequate precision.

- Recommendation: The authors should clarify whether the original RCT was powered to detect mediation effects. At minimum, a post hoc discussion of the sample size limitations should be added to the Study Limitations section.

Response 1

Thank you for your valuable suggestion. The following sentences have been added to clarify the sample size: *"The sample size calculation for the original trial was reported in a previous article.[15] While the primary trial was powered for 321 participants, but no formal sample size*

was calculated for the mediation analysis, which may have affected the statistical power of this analysis." (In the "Sample size" subsection of Methods, on page 3 (line 40-42) and page4 (line 1))

And we further elaborated on this limitation in the Discussion section: *"Second, the sample size was not determined based on the need for a causal mediation analysis, limiting the power of our analyses. But our analyses identified potential associations and provided a direction for future examination on potential mediation effects of vaccine confidence on uptake behaviors. We recommend that future vaccination behavioral trial designs could factor in vaccine confidence as potential mediators to further validate its role through collecting more robust trial data."* (In the Discussion section, page 12, lines 27-32)

Added reference

Li Y, Qin C, Qiu S, et al. The effectiveness of pay-it-forward in addressing HPV vaccine delay and increasing uptake among 15-18-year-old adolescent girls compared to user-paid vaccination: a study protocol for a two-arm randomized controlled trial in China. BMC Public Health. 2023;23(1):48.

(See Reference)

Comment 2: Use of the Vaccine Confidence Index (VCI)

- While the VCI is widely used, applying it in isolation—especially with a relatively modest sample size—may not capture the multifaceted nature of vaccine confidence in the context of HPV vaccination in China.

- Recommendation: The authors should justify their reliance on VCI in this study and acknowledge its reductive nature in the limitations section. Additional indicators or triangulated measures would have strengthened this construct.

Response 2

Thank you for highlighting the limitations of using the Vaccine Confidence Index (VCI) alone. we have added the following justification: *"Vaccine confidence was measured using the Vaccine Confidence Index (VCI)* [20]*, which centers on perception of vaccine safety, effectiveness, and importance. Its concise format enhances participant adherence. The VCI has demonstrated strong applicability in prior research on influenza vaccine confidence conducted in China.* [21,22]*"* (Lines 11–14 on page 4 of the Methods section)

In response to your suggestion, we have also acknowledged in the discussion section that: *"Finally, while the measures of vaccine confidence and delay intention were brief and feasible, they were somewhat narrow. Vaccine confidence was assessed solely using the VCI, and delay intention was defined mainly by vaccine type preference, potentially overlooking key factors such as risk perception, trust in healthcare, financial constraints, and social influences—limiting the ability to fully capture the complexity of vaccine decision-making."* (Lines 34-38 on page 12 of the Discussion section)

Added reference

Larson HJ, Schulz WS, Tucker JD, Smith DM. Measuring vaccine confidence: introducing a global vaccine confidence index. PLoS currents. 2015;7.

Wei Z, Sun X, Yang Y, Zhan S, Fu C. Seasonal influenza vaccine hesitancy profiles and determinants among Chinese children's guardians and the elderly. Expert Rev Vaccines. 2021;20(5):601-10.

Wu D, Jin C, Bessame K, et al. Effectiveness of a pay-it-forward intervention compared with user-paid vaccination to improve influenza vaccine uptake and community engagement among children and older adults in China: a quasi-experimental pragmatic trial. Lancet Infect Dis. 2022;22(10):1484-92.

(See Reference)

Comment 3: Inconsistencies Between Table 4 and Narrative Description

- Table 4 indicates that awareness of HPV infection, HPV vaccines, and vaccine types is higher among guardians of unvaccinated girls compared to those of vaccinated girls. However, the narrative in the Results and Discussion sections presents the opposite conclusion.

- Recommendation: This contradiction must be resolved. Either the table is mislabelled, the analysis misinterpreted, or the narrative needs to be corrected. This discrepancy directly affects the interpretation of vaccine-related knowledge as a barrier or enabler.

Response 3

We sincerely thank the reviewer for pointing out this important inconsistency. After carefully reviewing Table 4, the corresponding data analysis, and the related text in the Results and Discussion sections, we confirm that the table itself is correctly labeled and the analysis is accurate. The issue lies in the clarity of our narrative descriptions in the manuscript.

We have revised the text to provide a clearer and more accurate description in the Results section: *"The findings revealed that, among suburban participants, caregivers whose daughters were vaccinated through the program had lower baseline awareness of HPV and HPV vaccines those who chose not to vaccinate. (see Table 3). Specifically, prior to joining the project, 41.5% of vaccinated suburban participants did not know that the 2-valent vaccine was accessible in China, whereas only 21.1% of unvaccinated suburban participants lacked this awareness. Among urban participants, there was no statistically significant difference in pre-intervention vaccine knowledge between caregivers of vaccinated and unvaccinated girls. (P values > 0.05)"* (In the "Post hoc analysis" subsection of the Results, page 9, lines 20–27)

We also revised the Discussion section to reflect this finding and provide interpretation: *"It appears that caregivers with limited initial knowledge may be motivated to vaccinate once they receive relevant information, whereas those with greater prior awareness might still decline vaccination due to other factors such as cost, vaccine accessibility, preference for certain vaccine types, and prevailing social norms.[40-43] This highlights that vaccination decisions are influenced by a range of factors."* (In the Discussion section, page 12, lines 10-14)

Added reference

Lin Z, Chen S, Su L, et al. Influences of HPV disease perceptions, vaccine accessibility, and information exposure on social media on HPV vaccination uptake among 11,678 mothers with daughters aged 9-17 years in China: a cross-sectional study. BMC Med. 2024;22(1):328.

Vu M, Bednarczyk RA, Escoffery C, Ta D, Huynh VN, Berg CJ. U.S. Vietnamese parents' HPV vaccine decision-making for their adolescents: an exploration of practice-, provider-, and patient-level influences. J Behav Med. 2022;45(2):197-210.

Anandarajah A, Shato T, Humble S, et al. The association of caregiver attitudes, information sources, and trust with HPV vaccine initiation among adolescents. Human vaccines & immunotherapeutics. 2024;20(1):2300879.

Morgan JC, Badlis S, Head KJ, Zimet G, Cappella JN, Kornides ML. Types of HPV Vaccine Misinformation Circulating on Twitter (X) That Parents Find Most Concerning: Insights From a Cross-Sectional Survey and Content Analysis. Journal of medical Internet research. 2025;27:e54657.

(See Reference)

Comment 4: Interpretation of Mediation Effect Percentages

- The manuscript reports mediation percentages (e.g., 31.5%) but does not sufficiently explain how these were derived or what they imply in practical terms.

- Recommendation: The Discussion should offer a clearer interpretation of the mediation effect sizes, their public health relevance, and the assumptions under which they are valid.

Response 4

Thank you for pointing this out. The following sentences have been added:

We have added a detailed explanation in the Methods section: *"The proportion mediated was calculated by dividing the indirect effect by the total effect, indicating the extent to which the mediator accounts for the overall effect."* (Lines 29-30 on page 5 of the Methods section).

Furthermore, we have revised the Discussion section to provide a clearer interpretation of the mediation results and their public health implications: *"We found that vaccine delay intention may be an important mediator for the pay-it-forward behavioral intervention. Among urban populations, approximately 39% of the intervention's effect on HPV vaccination behavior was mediated through reduced vaccine delay intention. This suggests that addressing delay intentions—particularly the tendency to wait for the 9-valent vaccine—may be a key pathway through which behavioral interventions influence vaccination uptake. Interventions that enhance awareness of available vaccine options and encourage timely action within the optimal vaccination window may be especially effective in urban settings. Incorporating these elements into future health communication and service delivery strategies could help reduce vaccination delays and improve overall coverage."* (Lines 10–18 on page 11 of the Discussion section)

Comment 5 Oversimplification of Vaccine Delay Intention

- The study frames delay intention as being primarily driven by the availability of HPV vaccine types, which is an important but limited factor.

- Recommendation: The authors should acknowledge that this is a narrow representation of delay intention. Other known contributors—such as caregiver risk perception, trust in healthcare providers, financial burden, and perceived social norms—should be discussed even if not measured.

Response 5

Thank you for your thoughtful comment. We have added the following in the Methods section to explain the purpose of our definition of vaccine delay: *Given the high willingness of Chinese parents to vaccinate and their widespread preference for the 9-valent HPV vaccine—despite its limited supply—this study explored whether they would proceed with vaccination without delay if the preferred vaccine were easily accessible. To capture the specific Chinese context, a self-designed, scenario-based measure was used to assess participants' intention to delay vaccination.* (Lines 19-23 on page 4 of the Methods section)

Additionally, we have acknowledged this point in the Discussion section by adding the following sentence: "*However, we focused on vaccine type preference as a key reason for vaccine delay, while acknowledging that other important factors also influence vaccination decisions. Previous research indicated that vaccine delay is related to costs, lack of vaccine confidence, poor awareness of available vaccine products, and associated benefits.*[31-33]" (Lines 19–22 on page 11 of the Discussion section)

We also revised the Study Limitations section to reflect the constrained scope of our operational definition: *"Finally, while the measures of vaccine confidence and delay intention were brief and feasible, they were somewhat narrow. Vaccine confidence was assessed solely using the VCI, and delay intention was defined mainly by vaccine type preference, potentially overlooking key factors such as risk perception, trust in healthcare, financial constraints, and social influences—limiting the ability to fully capture the complexity of vaccine decision-making."* (Lines 34-38 on page 12 of the Discussion section)

Added reference

Sonawane K, Zhu Y, Montealegre JR, et al. Parental intent to initiate and complete the human papillomavirus vaccine series in the USA: a nationwide, cross-sectional survey. Lancet Public Health. 2020;5(9):e484-e92.

Zhao F, Qiao Y. Cervical cancer prevention in China: a key to cancer control. Lancet. 2019;393(10175):969-70.

Siu JY, Fung TKF, Leung LH. Social and cultural construction processes involved in HPV vaccine hesitancy among Chinese women: a qualitative study. Int J Equity Health. 2019;18(1):147.

(See Reference)

Comment 6 Mislabelled Odds Ratios in Table 2

- Table 2 refers to "OR" but reports adjusted odds ratios. This mislabelling should be corrected to "aOR" to reflect the multivariable model outputs and maintain terminological accuracy.

Response 6

Thank you for pointing out our error. We have carefully reviewed the entire manuscript and corrected all instances where "OR" was used to report adjusted odds ratios, replacing them with "aOR" to accurately reflect the multivariable model results.

VERSION 2 - REVIEW

**Reviewer**	**2**
**Name**	**RIAD, Abanoub**
**Affiliation**	**Masaryk University Faculty of Medicine, Department of Public Health**
**Date**	**29-Jun-2025**
**COI**	

Thank you for responding to my prior comments satisfactorily and amending your manuscript accordingly.

**Reviewer**	**3**
**Name**	**Dack, Kyle**
**Affiliation**	**University of Bristol, MRC Integrative Epidemiology Unit**
**Date**	**03-Aug-2025**
**COI**	**None**

Statistical review summary:

I have no major statistical concerns. Appropriate methods are used, and results are interpreted reasonably. I think the results could benefit from greater translation into actual clinical differences rather than focusing on the proportion of the mediation effect, but this is a minor improvement.

1. Title: I suggest dropping the question mark because there is not currently a question being asked in the title, and it makes "a causal mediation analysis?" sound uncertain.

Abstract:

2. Line 36-37. If there is space it would be helpful to clarify the intended meaning of the two mediators, in particular "vaccine delay intention" is not defined and could be interpreted in different ways.

3. Line 42-2[next page]: Is this comparing only those in a suburban setting? If so I suggest moving this modifier to the beginning of the sentence to improve clarity: “in those in a suburban setting, caregivers who vaccinated their children showed less prior awareness of HPV vaccination compared to those who did not”.

4. Line 42-2: The reported trend is “poorer prior awareness of vaccine”, but it may confuse readers because the poorer group has a higher percentage than the comparison group. This is because the percentages are not fully defined, and I suggest rephrasing to clarify and avoid misinterpretation, e.g. “a higher percentage of caregivers who vaccinated their children reported not being aware of the vaccine beforehand, compared to those who did not vaccinate their children (41.5% vs 21.1%[..]).

Introduction:

5. Very clear and informative – no comments.

Methods:

6. Generally, readability could be improved with more paragraphs when the topic changes, e.g. lines 14 (population and inclusion criteria) and Line 21 (randomization and procedure). I suggest also considering subheadings to further aid readers, there is no real downside to adding further subheadings to signpost relevant information.

7. The methodology is clear and comprehensive, including reasonable comments regarding lack of sample size calculation which is standard for secondary analyses, and the minimal confounder set. I have no concerns regarding these sections.

8. Statistical analysis: The analysis follows best practices and is reported very well, I have nothing further to suggest.

Results:

9. My only suggestion is that the reported proportion of mediating effects could be more directly translated into actual effect sizes, which would make it clearer to readers how significant these mediating factors were. E.g. vaccine delay intention mediated 28% of the total effect on vaccination, but what does this mean in terms of that individuals likelihood of vaccinating?

Discussion

10. No suggestions.

VERSION 2 - AUTHOR RESPONSE

Response to reviewer 3

Comment 1

Title: I suggest dropping the question mark because there is not currently a question being asked in the title, and it makes "a causal mediation analysis?" sound uncertain.

Response 1

Thank you for your valuable suggestion. In line with both your feedback and the journal's formatting requirements, we have removed the question mark and revised the title to eliminate the declarative style. The updated title is: "*Causal Mediation Analysis of a Randomized Controlled Trial in China: Evaluating Whether the Pay-It-Forward Strategy Increases HPV Vaccine Uptake by Reducing Vaccine Delay Intention and Increasing Vaccine Confidence*" (page 1, lines1-3)

Comment 2

Abstract: Line 36-37. If there is space it would be helpful to clarify the intended meaning of the two mediators, in particular "vaccine delay intention" is not defined and could be interpreted in different ways.

Response 2

Thank you for your helpful comment. To enhance clarity, we have added brief definitions of both mediators in the abstract. The revised text reads: *"Based on previous literature, we hypothesized that vaccine confidence and vaccine delay intention were potential mediators. Vaccine confidence was measured using the Vaccine Confidence Index (VCI). Vaccine delay intention refers to the caregiver's preference to postpone HPV vaccination for their daughter until the preferred vaccine type becomes available, rather than accepting the immediately accessible HPV vaccine. Data on these mediators were collected via a self-administered online questionnaire conducted after the intervention but before vaccination."* (In the Abstract section, page 1, lines 35-41)

Comment 3

Abstract: Line 42-2[next page]: Is this comparing only those in a suburban setting? If so I suggest moving this modifier to the beginning of the sentence to improve clarity: "in those in a suburban setting, caregivers who vaccinated their children showed less prior awareness of HPV vaccination compared to those who did not".

Response 3

Thank you for your suggestion. We have revised the sentence by moving the setting modifier to the beginning for clarity. The revised sentence reads: "*In the suburban setting, a higher percentage of caregivers who vaccinated their daughters reported not being aware of the HPV vaccine before participating in the trial, compared to those who did not vaccinate their daughters (41.5% vs 21.1%; P = 0.011).*" (In the Abstract section, page2, lines 4-6)

Comment 4

Abstract: Line 42-2: The reported trend is "poorer prior awareness of vaccine", but it may confuse readers because the poorer group has a higher percentage than the comparison group. This is because the percentages are not fully defined, and I suggest rephrasing to clarify and avoid misinterpretation, e.g. "a higher percentage of caregivers who vaccinated their children reported not being aware of the vaccine beforehand, compared to those who did not vaccinate their children (41.5% vs 21.1%[..]).

Response 4

Thank you for pointing this out. We agree that the original phrasing could be misleading.We have revised the sentence to clarify that the percentages refer to the proportion of caregivers who were not aware of the vaccine prior to the trial. The updated sentence is: *"In the suburban setting, a higher percentage of caregivers who vaccinated their daughters reported not being aware of the HPV vaccine before participating in the trial, compared to those who did not vaccinate their daughters (41.5% vs 21.1%; P = 0.011)."* (In the Abstract section, page2, lines 4-6)

Comment 5

Methods: Generally, readability could be improved with more paragraphs when the topic changes, e.g. lines 14 (population and inclusion criteria) and Line 21 (randomization and procedure). I suggest also considering subheadings to further aid readers, there is no real downside to adding further subheadings to signpost relevant information.

Response 5

Thank you for your suggestion. We have revised the Methods section to improve readability by breaking up the text into shorter, topic-specific paragraphs. Additionally, we have added subheadings such as "Study Design," "Population and Inclusion Criteria," "Randomization and Procedure," and "Intervention" to better guide the reader through the methodology.. (In the Methods section, page 3, lines 9-33)

Comment 6

Results: My only suggestion is that the reported proportion of mediating effects could be more directly translated into actual effect sizes, which would make it clearer to readers how significant these mediating factors were. E.g. vaccine delay intention mediated 28% of the total effect on vaccination, but what does this mean in terms of that individuals likelihood of vaccinating?

Response 6

Thank you for this helpful suggestion. We have revised the Results section to provide a clearer interpretation of the mediating effect in terms of their practical significance. Specifically, we have added the following: "This finding suggests that reducing vaccine delay intention

accounted for about 39% of the intervention's effect on HPV vaccination among urban participants and 28% of the effect in the overall population." (In the Results section, page 9, lines 4-6)
